# DNA methylation biomarkers of intellectual/developmental disability across the lifespan

**DOI:** 10.1186/s11689-025-09598-5

**Published:** 2025-02-19

**Authors:** Janine M. LaSalle

**Affiliations:** Department of Medical Microbiology and Immunology, Perinatal Origins of Disparities Center, MIND Institute, Genome Center, Environmental Health Sciences Center, University of California Davis, One Shields Ave., Davis, CA 95616 USA

**Keywords:** DNA methylation, Epigenetics, Autism, Down syndrome, Dup15q syndrome, Exposure, Genomic, Epigenetic clock, Aging, Biomarkers, Placenta, Cord blood, Cell free DNA

## Abstract

Epigenetic mechanisms, including DNA methylation, act at the interface of genes and environment by allowing a static genome to respond and adapt to a dynamic environment during the lifespan of an individual. Genome-wide DNA methylation analyses on a wide range of human biospecimens are beginning to identify epigenetic biomarkers that can predict risk of intellectual/developmental disabilities (IDD). DNA methylation-based epigenetic signatures are becoming clinically useful in categorizing benign from pathogenic genetic variants following exome sequencing. While DNA methylation marks differ by tissue source, recent studies have shown that accessible perinatal tissues, such as placenta, cord blood, newborn blood spots, and cell free DNA may serve as accessible surrogate tissues for testing epigenetic biomarkers relevant to understanding genetic, environmental, and gene by environment interactions on the developing brain. These DNA methylation signatures may also provide important information about the biological pathways that become dysregulated prior to disease progression that could be used to develop early pharmacological interventions. Future applications could involve preventative screenings using DNA methylation biomarkers during pregnancy or the newborn period for IDDs and other neurodevelopmental disorders. DNA methylation biomarkers in adolescence and adulthood are also likely to be clinically useful for tracking biological aging or co-occurring health conditions that develop across the lifespan. In conclusion, DNA methylation biomarkers are expected to become more common in clinical diagnoses of IDD, to improve understanding of complex IDD etiologies, to improve endpoints for clinical trials, and to monitor potential health concerns for individuals with IDD as they age.

## Background

There has been incredible progress in understanding the genetic basis of intellectual/developmental disabilities (IDD) over recent decades following the sequencing of the human genome. With recent advances in whole exome and whole genome sequencing, over 1500 novel IDD genes have been identified and diagnostic yield for identifying causative mutations is 30–35% [1]. Genetic tests are the most relevant of all laboratory biomarkers because they define specific IDD syndromes in which most symptoms can be predicted based on genotype. However, there are limitations to exclusively using the predictive promise of genotype-forward testing approaches. Specifically, the etiologies of many IDDs are complex, involving a combination of inherited and sporadic variants of multiple genes, as well as gene by environmental interactions across life stages. Even for characterized genetic IDD syndromes with high penetrance, there can be large differences between individuals within the same syndrome in their clinical presentation. Thus, there is a distinct need to go beyond the simplicity of the four nucleotide DNA code in genetic testing to develop clinically actionable predictive biomarkers through epigenetics.

Epigenetics literally means “on top of” genetics. Epigenetic mechanisms are defined as changes to nucleotides or chromatin that do not change the DNA sequence but can alter gene expression and resulting phenotypes. Epigenetics includes multiple layers on top of DNA, including DNA methylation, histone modifications, chromatin accessibility and organization, and chromatin-bound noncoding RNAs. Epigenetic mechanisms are particularly important for mammals because of their prolonged developmental period and long life spans, prompting the need for ways to adapt a static genome to a dynamic environment across life stages and potentially across generations. Large brain sizes in primates prompted the need for sophisticated homeostatic mechanisms regulating energy and oxygen supplies to support the excessive needs of fetal brain development. Remarkably, the developing brain uses roughly 60% of the fetus’ energy and oxygen supplies, despite being only 13% of body weight [2]. At the interface of genetic and environmental contributions to brain development and function, DNA methylation marks can be thought of as fossil footprints left in the complex and dynamic networks of how genes and their non-genetic regulators responded to multiple challenges during human brain development. DNA methylation is also the epigenetic layer most amenable to testing in a variety of human biospecimens and most influenced by DNA sequence. As DNA methylation changes can also influence gene expression, they can be directly involved in the disease pathogenesis of specific IDDs and provide important clues about downstream dysregulated gene networks that could be targeted for novel therapies.

The term “biomarker” has been defined as “characteristics measured as indicators of normal biological processes, pathogenic processes, or responses to an exposure or intervention, including therapeutic interventions” [3]. Molecular biomarkers have been classically considered to be quantitative tests of individual genes or molecules. However, discoveries from unbiased approaches involving -omic technologies combined with predictive algorithms have resulted in a new generation of “signature” molecular biomarkers [4, 5]. An “epigenetic signature” refers to a specific set of DNA methylation differences that have been selected to discriminate IDD from typically developing DNA samples by unsupervised learning approaches [6, 7]. Like the flexibility in recognition of a handwritten signature, a well-supported epigenomic signature does not need to be replicated precisely at every methylated site to be accurate. Instead, it is the combination of methylation changes that is collectively predictive, an approach that is more forgiving of biological noise and confounding factors than that observed with measuring a single methylated site. While these epigenomic signatures are frequently secondary events in the disease pathogenesis downstream of the causative mutation or exposure, they hold promise in improving disease prediction as well as providing molecular insights into disease etiology, molecular pathogenesis, and treatment.

This review will focus on DNA methylation biomarkers identified through genome-wide approaches, including both microarrays and sequencing-based approaches. Although biomarkers are classically considered not to require a known biological function to be useful, epigenetic biomarkers have provided important insights into convergent gene networks between different syndromic and idiopathic IDDs that include many of the most “druggable” gene targets. Furthermore, the promise of biomarkers to reveal insights into elusive gene by environmental interactions in IDDs will also be reviewed and discussed. Lastly, I will discuss where the field is going into the next frontier of predicting and monitoring IDDs through epigenomics and machine learning.

## Technical and biological challenges influencing DNA methylation patterns in human biospecimens

A major consideration in the search for epigenetic biomarkers of IDDs is which other factors or technical variables are influencing DNA methylation patterns that may confound or affect the interpretation of IDD-associated differential methylation. The most important consideration is that many (but not all) DNA methylation patterns are cell- and tissue-specific. This feature is an advantage because cell type-specific DNA methylation sites have been identified that are so unique that they can serve as identifiable bar codes that can identify proportions of cell types within tissues [8]. Thus, cell type adjustment approaches are now standard for epigenome-wide association studies (EWAS) analysis pipelines in commonly used human tissues such as blood. The potential disadvantage to the cell type specificity of DNA methylation is the assumption that if you are looking for epigenetic biomarkers of disorders that affect the brain, these will be hard to identify in blood or other peripherally accessible tissue. However, as will be evident in more detail in the examples below of IDD-specific DNA methylation signatures identifiable in blood and placenta, these “surrogate” tissues can often be useful as accessible windows into the epigenetic changes that have also occurred within the inaccessible brain [9–11].

Age is another major factor that can impact DNA methylation patterns. This finding has spawned a new field of “epigenetic clock” calculations of accelerated aging through the comparison of epigenetic to chronological age [12]. There are now multiple age calculators based on DNA methylation array data from human populations of different age ranges. Adult epigenetic clocks are predictive of all-cause mortality in later life [13]. In addition, accurate gestational epigenetic age calculators have been developed for newborn cord blood and placenta [14, 15]. Contrary to the associations of elevated epigenetic age in adults with a variety of environmental stressors, deceleration in epigenetic gestational age is predominantly observed with adverse prenatal factors, indicating that development was delayed. As an example, prenatal adverse environment measured by decreased cerebroplacental ratio in the third trimester was associated with epigenetic age deceleration and decreased cord blood methylation of the EP300 gene involved in hypoxia and schizophrenia genetic liability [16]. In adulthood, epigenetic age acceleration was associated with reductions in cognitive processing speed in middle age [17, 18], suggesting that epigenetic age biomarkers could be useful for monitoring health across the lifespan in individuals with IDD.

Sex is another important biological variable to consider for epigenetic biomarkers, largely because of the influence of X chromosome inactivation specifically in females [19], but also because of the impact of sex on epigenetic marks at autosomal loci [20]. Since neurodevelopmental disorders have a strong male bias, sex is also critically important for understanding the underlying biology behind the sex differences in susceptibility [21]. While most array-based EWAS studies simply eliminate the sex chromosomes from analyses, this is problematic because the X chromosome is significantly enriched for genes expressed in the nervous system [10] and mutated in IDDs [22]. Recent bioinformatic pipelines have been developed for analyzing DNA methylation sequencing and array data that includes the X and Y chromosomes [23]. Comparing sex-adjusted versus sex-stratified analyses is also recommended, as was insightful inidentifying differentially methylated regions (DMR) associated with autism spectrum disorders (ASD) from newborn blood collected prior to diagnosis [10]. Despite female samples being underrepresented four-fold in ASD biospecimens, sex stratification resulted in greater power to detect informative DNA methylation patterns in females than the usual approach of combining both sexes in the analyses and adjusting for sex as a confounding variable. For example, in the X-linked dominant Rett syndrome (MIM#312750), caused by heterozygous mutations of *MECP2* in females, sex differences in disease models go beyond what is expected based on gene dosage alone [24, 25]. Even for syndromic forms of IDD caused by autosomal gene mutations in humans and mouse models, disease severity and phenotypes can show profound sex differences [26, 27].

## Clinical utility of DNA methylation signatures of genetic syndromic IDDs

Genetic differences can highly impact DNA methylation signatures, both in *cis* and in *trans*. *Cis* effects include both variants that directly change CpG sites or single nucleotide variants (SNVs) that have been determined to be methylation quantitative trait loci (mQTL) to neighboring genes [28, 29]. A recent nanopore sequence study of long-read DNA haplotypes in blood confirmed that DNA sequence variability in *cis* explains a large part of the correlation found between gene expression and CpG methylation [30]. Their results were equally consistent with two proposed *cis* models: one in which sequence variation affects the binding of transcription factors (TF) as an intermediary to DNA methylation differences versus one in which variants directly impact DNA methylation patterns.

**Fig. 1:**
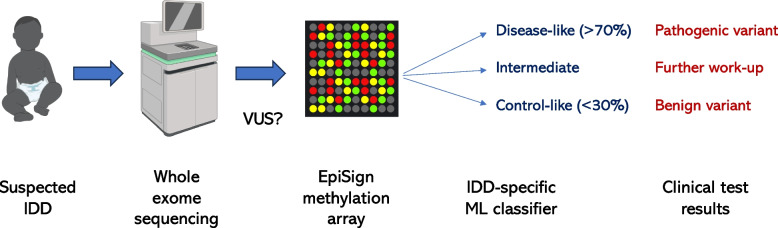
Epigenetic signatures provide clinically useful information following exome sequencing. EpiSign is a classifier of DNA methylation signatures of genetic syndromic forms of IDD, particularly chromatinopathies. Variants of unknown significance (VUS) from exome sequencing results can be followed up with EpiSign to help determine if a variant is pathogenic, benign, or requires further molecular work-up to determine why an intermediate epigenetic signature is observed. BioRender.com was used for some images

Demonstrating the utility of DNA methylation in clinical genetics, genome-wide epigenetic signatures in *trans* have been reported for over 50 different syndromic forms of IDD that are observed in peripheral blood DNA from Illumina Infinium arrays [31–33]. The use of DNA methylation signatures for identifying and characterizing syndromic IDDs, also known as EpiSign, is an emerging and promising direction in clinical genetics (Fig. 1). EpiSign methylation array data combined with machine learning classifiers trained on known syndromic forms of IDD are being utilized to follow-up on non-definitive results from whole exome sequencing. Specifically, EpiSign classifiers can help determine if variants of unknown significance (VUS) in suspected syndromic genes are pathogenic, benign, or intermediate in the downstream molecular phenotypes. In a cohort of 207 subjects referred for genetic testing, 136 patient samples had a previous VUS finding, and of these, 35.3% had DNA methylation profiles positive for one of the EpiSign classifiers [34].

Most of the IDD syndromes with clear epigenomic signatures detected on DNA methylation arrays are classified as “chromatinopathies” because they involve mutations to genes encoding epigenetic machinery or other transcriptional regulators. Examples of those with the greatest sensitivity for VUS classification in IDDs include Kabuki syndrome (*KMT2D,* MIM#147920), alpha-thalassemia/mental retardation syndrome (*ATRX*, MIM#301040), Tatton-Brown-Rahman syndrome (*DNMT3A*, MIM#615879)*,* and Sotos syndrome (*NSD1*, MIM# 117550) [33, 35]. In addition, overlap between some aspects of DNA methylation signatures can be informative about convergent molecular pathways of IDD pathogenesis and potential therapies. As an example, the hypomethylated signature specific to intergenic regions in common to both Sotos [36] and Tatton-Brown-Rahman [37] syndromes was found to be due to the loss of H3K36me2 epigenetic marks made by the histone methylase NSD1 that are critical for recruitment of the DNA methyltransferase DNMT3A to transcriptionally active intergenic regions in development [38]. Furthermore, different chromatinopathies caused by mutations in histone methyltransferases genes have been shown to exhibit either accelerated (*NSD1*, Sotos syndrome) or decelerated (*KMT2D,* Kabuki syndrome) epigenetic aging, as determined by DNA methylation array signatures [37].

Other IDDs with clear epigenomic signatures are aneuploid (Down syndrome, trisomy 21) or copy number variants (CNVs, including 7q11.23 duplication, 7q11.23 deletion, 15q11.2–13.3 duplication, 16p11.2 deletion, or 17q23.1-q24.2 duplication syndromes) [32, 39]. Use of epigenomic signatures can be clinically useful for genotype–phenotype correlations in IDD patients with atypical CNVs that are smaller deletions or duplications than those that define the syndrome. For instance, atypical CNV cases revealed that *GTF2I* genes, encoding transcription factors, were the major contributor to the distinct DNA methylation signatures of 7q11.23 duplication and 7q11.23 deletion cases [40]. In addition to the pre-genomic era knowledge that maternal DNA methylation of the imprinting control region is diagnostic for 15q11.2–13.3 duplication syndrome [41], whole methylome sequencing analyses of postmortem brain samples revealed a distinct hypomethylation signature of gene bodies with functions in neuronal synapses [42]. A later comparison of DNA methylation signatures in postmortem brain between Dup15q, Rett syndrome, and idiopathic ASD revealed largely independent epigenetic signatures, but with convergent differentially methylated genes also known to be mutated or differentially expressed in ASD brain [43].

Down syndrome (DS), caused by trisomy 21, has not been categorized as either a chromatinopathy or a chromosome characterized by epigenetic influences of parental imprinting. Yet, multiple studies have demonstrated DNA methylation signatures in DS tissues occur genome-wide rather than enriched on chromosome 21, suggesting *trans* effects of the trisomy [44–49]. Two chromosome 21 encoded genes have been implicated in the trans effects of trisomy 21 on other chromosomal methylation patterns [44–51]. *DNMT3L* encodes a DNA methyltransferase and its overexpression in a neuronal cell line recapitulated much of the hypermethylated signature of DS [51]. The developmental transcription factor *RUNX1*, also encoded on chromosome 21, was identified within the transcription factor motifs of DS hypomethylated loci [46]. Furthermore, the newborn blood DNA methylation signature of DS can further differentiate DS cases with and without congenital heart defects when sex was considered in the analyses of differentially methylated regions [52]. With > 700 human genes encoding chromatin and transcriptional regulators [53], it is perhaps not surprising that an extra copy of an entire chromosome includes one or more genes that when overexpressed will leave a detectable footprint on the methylome patterns of other chromosomes. Therefore, studies on the epigenetics signature of DS illustrate an important point: functional studies of IDDs resulting from CNVs should not necessarily be limited to genes within each CNV, but also consider the impact of each candidate gene in *trans*.

## Epigenetic biomarkers of environmental exposures associated with IDD

Environmental exposures include what is recently referred to as the “exposome” that includes the entirety of nongenetic factors influencing human disease risk. These include chemical (heavy metals, pesticides, cosmetics), physical (climate, light, radiation), and biological (viral, microbial, fungal) exposures, as well as socioeconomic (poverty, inequality, cultural norms), lifestyle (nutrition, smoking, drug use), and psychological (stress, depression, anger) factors [54]. A major challenge to understanding how different aspects of one’s exposome impact disease risk include the difficulty in measuring exposures after a disease has been diagnosed. Furthermore, not all individuals respond the same way to the same level of a specific exposure. Acting at the interface between genetics and environmental exposures, DNA methylation biomarkers of exposures have the potential for deconvolving the complexity of an individual’s exposome in IDD risk and disease progression [55].

In addition to genetic factors, multiple environmental exposures have been implicated in the development of IDD. The evidence is particularly strong for maternal factors during pregnancy (infection, malnutrition, smoking, alcohol, obesity, preeclampsia) and birth complications (hypoxia, prematurity, small for gestational age) [22]. Exposure to specific environmental chemical and IDD risk have been extensively investigated and include heavy metals, persistent organic pollutants, and maternal smoking [54, 55]. For array-based blood EWAS, DNA methylation sites that were the most reproducible for maternal exposures included smoking, folate, dietary glycemic index, air pollution, and metals [56]. In the interest of focusing on epigenetic biomarkers, this section will focus on those that have the most reproducible DNA methylation signatures associated with IDDs, maternal smoking, folic acid, and lead.

Prenatal smoking has been associated with a reproducible epigenetic signature across a meta-analysis of 13 cohorts within the Pregnancy And Childhood Epigenetics (PACE) consortium [57]. Of the five genes differentially methylated with exposure to maternal smoking, *DLGAP2* (discs large homolog-associated protein 2) is of particular importance for IDD, since rare variants in *DLGAP2* were identified in progressive epilepsy with mental retardation (EPMR [MIM#610003) [58] as well as ASD [59]. Folic acid supplementation through prenatal vitamin use in the first month of pregnancy has been associated with a reduced neural tube defects as well as ASD. DNA methylation differences in newborn blood from mother’s folic acid use mapped to 320 genes, including *CSDM2* [60], a regulator of innate immune responses in the brain mutated in IDD with cortical malformations [61]. Two recent studies of prenatal lead exposure and DNA methylation in cord blood differed in the top genes identified and in the sex bias in number differentially methylated probes identified [62, 63]. However, a neurodevelopmentally relevant gene replicated across both studies, *GPR155*, encoding a G protein-coupled receptor acting through the mTOR signaling pathway that has been shown to be a hub for transcriptional dysregulation in ASD brain [64].

## Epigenetic biomarkers of gene by environmental interactions associated with IDD

Gene by environmental interactions (GxE) can explain how the impact of a specific exposure on disease risk is different between people of different genotypes. Or vice-versa, the impact of a particular genotype on disease risk is different depending on environmental exposures. At the interface of GxE, DNA methylation studies have attempted to estimate the influence of GxE on variance of DNA methylation patterns in newborns. One array-based study estimated that 75% of variably methylated regions in cord blood were explained by genotype x in utero environment interactions, compared to only 25% by genotype alone [28]. A larger study concluded that genetic, gene plus environment (G + E), and GxE explained roughly equal proportions of variably methylated regions, and that ASD was the neurologic disorder best explained by GxE [65].

Using the logic and language of epidemiology, DNA methylation differences associated with disease can be further investigated for specific mechanisms in the disease etiology [66]. Namely, DNA methylation changes may directly mediate the effects of environment or genotype on disease pathogenesis. Alternatively, DNA methylation may modify the degree to which the environmental or genetic factors impact disease risk. Lastly, DNA methylation patterns influenced by GxE may be indirectly involved in the mechanism of disease through their impacts on gene regulation. However, to be useful as biomarkers, DNA methylation markers do not need to be mechanistically involved. This is especially true when DNA methylation differences are multiplexed as epigenomic signatures, since the main goal is that they are predictive of disease or severity. But several recent studies described below have suggested that DNA methylation signatures can reveal novel insights into the complex mechanisms in GxE in IDD.

As an example, 15q11-q13 duplication is a syndromic form of IDD (Dup15q) and is one of the most common CNVs identified in ASD [41]. Dup15q syndrome results from a duplication that is either extrachromosomal or interstitial, but ASD is only observed when the interstitial duplication is maternal in origin, due to the imprinted gene *UBE3A* [67]. Surprisingly, in our prior measurements of polychlorinated biphenyl (PCB) and polybrominated diphenyl ethers (PBDE) within human postmortem brain samples, Dup15q syndrome was the highest predictor of PCB 95 levels, not idiopathic ASD, as hypothesized [68]. We further identified the epigenomic signatures of the interaction with PCB 95 in both postmortem brain and a neuronal cell line model of Dup15q syndrome [42, 69]. DNA hypomethylated genes with functions in neuronal synapses were identified in Dup15q cortex with PCB 95 exposure, and the long-term neuronal cultures in PCB 95 showed a significant overlap with the DNA methylation changes [42]. These results supported a multi-hit model of convergent gene pathways between these genetic and environmental factors. We also explored a possible biochemical mechanism for the overlap, showing that the UBE3A target RING1B is a ubiquitin ligase for the histone components H2A and H2A.Z [70]. Ubiquitinated H2A.Z, a poised developmental mark of large chromatin domains, correlated with lower levels of DNA methylation, and PCB 95 independently reduced the levels of H2A.Z in the cell culture model [42].

In other examples of using DNA methylation signatures to investigate GxE interactions, we examined the influence of prenatal vitamin use on DNA methylation in placenta from the prospective Markers of Autism Risk in Babies – Learning Early Signs (MARBLES) study. This was prompted by that fact that in several epidemiological studies, mothers who took a prenatal vitamin in the first month of pregnancy showed a lower likelihood of having a child with ASD [71, 72]. In a pilot study of placenta samples collected before the diagnosis of 20 ASD compared to 21 typically developing (TD) offspring, differential methylation of two genes, *CYP2E1* and *IRS2*, were validated and examined for associations with common genotypes and prenatal vitamin use [73]. Prenatal vitamin use (folic acid supplementation) in the first pregnancy month was significantly associated with lower methylation at *IRS2* in a direction consistent with protection from ASD. Furthermore, sixty additional genes were in common between those differentially methylated with prenatal vitamin use and those differentially methylated in ASD placenta [73]. In a more recent study of 204 placental samples from two high risk ASD cohorts we identified a novel neuroprotective gene locus that we named neuronal hypoxia inducible, placenta associated (*NHIP*) [9]. A common structural variant near a putative *NHIP* enhancer was a better predictor of ASD in these cohorts than polygenic risk score for ASD in this cohort. However, use of a prenatal vitamin in the first month of pregnancy appeared to counteract the genetic likelihood of ASD at the *NHIP* locus through higher DNA methylation [9]. Together, these studies suggest that a simple dietary folic acid intervention, if started prior to becoming pregnant, can be an inexpensive modifier of ASD genetic likelihood, and that epigenetic biomarkers may help in the monitoring of intervention efficacy.

## Future directions in epigenetic biomarkers: optimism tempered by ethical considerations and limitations

**Fig. 2:**
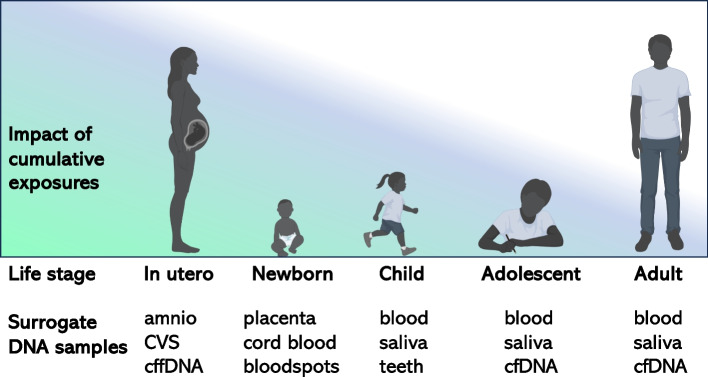
Potential for epigenetic biomarkers for IDDs from accessible tissue sources across the lifespan. Color gradient represents the higher impacts of cumulative exposures on IDD and epigenetic changes in utero and early life. Several accessible “surrogate” tissues, some specific to distinct life stages, can be used for DNA methylation analyses and biomarkers that reflect brain and cognition as well as other health conditions that develop across the life course. BioRender.com was used for some images

The future of epigenetic biomarkers is expected to continue its promising trajectory. Despite the inherent limitations of current epigenome-wide association studies, sample sizes and technologies are expected to continue to improve biomarker discovery. With the continued decrease in DNA sequencing costs and improved computational pipelines, sequencing-based technologies are expected to replace hybridization-based arrays [7]. While brain DNA is largely inaccessible in living humans, several surrogate DNA sources are likely to be important for monitoring DNA methylation patterns relevant to brain health across the lifespan (Fig. 2). Replication across multiple different cohorts with diverse ancestries and socioeconomic demographics will be critical in developing the current signature-based screens into more robust biomarkers.

Cell free DNA (cfDNA) can be isolated from blood plasma at any age. But during pregnancy, maternal plasma contains cfDNA derived from maternal blood cells as well as cell free fetal DNA (cffDNA) derived from trophoblast cells of the placenta [74, 75]. In early pregnancy, cffDNA is tested for genetic aneuploidies and large CNVs, thereby enabling a pre-screen for the likelihood of IDDs including Down syndrome and Dup15q syndrome. Confirmation of the diagnosis of genetic IDDs is performed by chorionic villus sampling (CVS) or amniocentesis. Currently epigenetic testing of cfDNA is being developed for multiple cancer detection in adults [76, 77], as similar to placenta, tumors shed their DNA into circulating blood. In the future, epigenetic testing of cffDNA during pregnancy may be useful for predicting the diagnosis or severity of IDDs. Prenatal DNA methylation testing may be most effectively introduced for pregnancies at high risk for IDDs based on genetic or non-genetic risk factors and potentially combined with current genetic screens. However, improved accuracy of DNA methylation biomarkers would be necessary before adding these to existing genetic prenatal screening because of potential parental stress and possible termination resulting from false positives. An additional ethical consideration would be the potential mental distress to the pregnant person in feeling blame or stigmatization due to epigenetic biomarkers reflective of lifestyle factors [78].

Newborn screening is another area that seems promising for the development of epigenetic biomarkers. Both placenta and cord blood are accessible at birth and newborn blood spots are routinely collected for state-based metabolic screening programs [79]. Identifying newborns with epigenetic signatures of IDD-predictive biomarkers and/or gene x environmental interactions could result in earlier interventions and improved developmental trajectories in future generations. To save costs, such a screening could be limited to high-risk pregnancies and births that involve fetal or newborn hypoxia or other medical complications [80]. Later in childhood, another novel resource is shed baby teeth that can be collected during childhood for the study of cells, DNA, and exposures associated with IDDs [81, 82]. Dental pulp stem cells have been utilized to investigate epigenetic differences in Dup15q syndrome, for example [83].

Lastly, adolescents and adults with IDD continue to have health concerns throughout their lifespans that may benefit from epigenetic biomarker monitoring. The epigenetic clock assays could provide information on the rate of biological aging, which may be accelerated or decelerated in some genetic syndromes [37, 84], depending on the life stage. Cancer screens using cfDNA may be especially important for syndromic IDD, as many of these genes share overlap with cancer risk [85]. Other systems, including cardiovascular, pulmonary, and immune are likely to help in precision health care for adults with IDD. Lastly, prediction of dementia, including in Down syndrome individuals who are already genetically susceptible to Alzheimer’s [86], may be possible in the future with epigenetic biomarkers of early pre-symptomatic stages.

There are certainly ethical considerations that will arise from a broader clinical use of DNA methylation biomarkers in health care decisions [87, 88]. On the positive side, DNA methylation patterns reflect the complex interplay between genetic predisposition and nongenetic resiliency factors, pointing to novel avenues for prevention and early intervention rather than strict genetic determinism. On the negative side, the emphasis on lifestyle factors that influence DNA methylation could tilt the blame towards the individual’s responsibility rather than the “bad luck” that is usually ascribed to genetic mutations. For improving the lives of individuals with IDDs, the hope is that DNA methylation biomarkers will serve to complement existing genetic tests in initial diagnoses, improve quantifiable endpoints of clinical trials, and monitor health challenges across the lifespan, including those with aging.

## Conclusions

Recent studies of DNA methylation in human IDD cohorts support the premise that epigenetic marks hold great promise for development of future biomarkers. Advances in epigenetic biomarkers are expected to be important in reducing diagnostic disparities in IDD, including ASD, as well as for monitoring therapeutic interventions throughout the lifespan.

## Data Availability

No datasets were generated or analysed during the current study.

## Abbreviations

Amnio: Amniocentesis
ASD: Autism spectrum disorder
cfDNA: Cell free DNA
cffDNA: Cell free fetal DNA
CNV: Copy number variant
CVS: Chorionic villus sampling
DMR: Differentially methylated regions
DNA: Deoxyribonucleic acid
DS: Down syndrome
EWAS: Epigenome-wide association study
IDD: Intellectual/developmental disability
MARBLES: Markers of Autism Risk in Babies – Learning Early Signs
mQTL: Methylation quantitative trait loci
NHIP: Neuronal hypoxia inducible, placenta associated
PCB: Polychlorinated biphenyl
PBDE: Polybrominated diphenyl ethers
RNA: Ribonucleic acid
SNVs: Single nucleotide variants
TF: Transcription factors
TD: Typically developing
VUS: Variant of unknown significance
